# Retrospective analysis of doxorubicin and prednisone as first-line therapy for canine B-cell lymphoma

**DOI:** 10.1186/s12917-018-1688-5

**Published:** 2018-11-20

**Authors:** Sami Al-Nadaf, Robert B. Rebhun, Kaitlin M. Curran, Rachel O. Venable, Katherine A. Skorupski, Jennifer L. Willcox, Jenna H. Burton

**Affiliations:** 1 William R. Pritchard Veterinary Medical Teaching Hospital, School of Veterinary Medicine, University of California, Davis, Davis, CA USA; 2 Department of Surgical and Radiological Sciences, School of Veterinary Medicine, University of California, Davis, Davis, CA USA; 3 Department of Clinical Sciences, Carlson College of Veterinary Medicine, Oregon State University, Corvallis, OR USA; 4 Arizona Veterinary Oncology, Gilbert, AZ USA

**Keywords:** Adriamycin, Cancer, Canine, Chemotherapy, Immunophenotype

## Abstract

**Background:**

A doxorubicin (DOX)-based chemotherapy protocol, CHOP, is the most effective treatment for canine high-grade B-cell lymphoma; however, the cost and time requirements associated with this protocol are not feasible for many pet owners. An alternative treatment option is the use of DOX, the most effective drug, in combination with prednisone. Prior studies with single-agent DOX included dogs with T-cell lymphoma, a known negative prognostic factor, which may have resulted in shorter reported survival times than if dogs with B-cell lymphoma were analyzed separately. The purpose of this study was to evaluate the outcome of dogs with high-grade B-cell lymphoma when treated with DOX and prednisone with or without L-asparaginase (L-ASP). Identification of prognostic factors was of secondary interest.

**Results:**

Thirty-three dogs were included in the study; 31 dogs were evaluable for response with an overall response rate of 84%. The median progression free survival (PFS) and overall survival (OS) were 147 days and 182 days, respectively. The one-year survival fraction was 23%. No variable other than protocol completion was found to be significant for either PFS or OS including historical prognostic factors such as substage, thrombocytopenia, and body weight.

**Conclusions:**

Dogs with high-grade B-cell lymphoma treated with DOX and prednisone with or without L-ASP have similar response rates, PFS, and OS to prior studies that did not differentiate between lymphoma immunophenotype. This protocol is not a replacement for CHOP; however, it is an alternative if time and cost are factors, while providing therapeutic benefit greater than prednisone alone.

## Background

A doxorubicin (DOX)-based chemotherapy protocol including cyclophosphamide, vincristine, and prednisone (CHOP), with or without L-asparaginase (L-ASP), is the most effective treatment for canine high-grade lymphoma. CHOP has repeatedly shown remission rates greater than 85%, with response durations of 6 to 10 months and survival times of 8 to 12 months [1–5]. However, several studies have described distinct treatment outcomes between high-grade B- and T-cell lymphoma demonstrating immunophenotype as an important prognostic factor [6–8]. When treated with a CHOP protocol, dogs with high-grade T-cell lymphoma have significantly shorter remission and survival times compared to those reported for dogs with high-grade B-cell lymphoma [9]. Multi-agent protocols report similar response rates for dogs with B- and T-cell lymphoma [10, 11]. However, response rates to a single dose of DOX for dogs with T-cell lymphoma is 50% compared to a 100% response rate for dogs B-cell lymphoma, suggesting that DOX may be a less efficacious drug for the treatment of T-cell lymphoma in dogs [12]. When deciding on a course of therapy for a dog with lymphoma, many owners consider prognosis as well as cost and time commitment of treatment. The financial burden and time requirement to pursue a CHOP protocol is not feasible for many pet owners, necessitating alternative options when CHOP is declined.

DOX is the most effective single-agent drug for the treatment of canine high-grade lymphoma and DOX combined with prednisone may be offered as an alternative option to CHOP [13, 14]. This may be a more practical treatment for owners seeking to extend quality of life beyond that obtained with prednisone alone, but who are interested in a less intense protocol in terms of frequency and total number of treatments as well as cost [9]. When administered as a single-agent protocol, DOX is typically administered once every 3 weeks for 5 to 6 treatments whereas a CHOP protocol may consist of 12 to 17 chemotherapy treatments administered over 15 to 26 weeks. Outcome of dogs with lymphoma treated with a single-agent DOX protocol is inferior to CHOP, with shorter remission durations (4 to 6 months) and decreased survival times (5 to 9 months) despite relatively similar response rates (74 to 88%) [15–19]. However, previous studies evaluating DOX administered every 3 weeks for 5 to 6 treatments have not examined the association of patient outcome and immunophenotype. This raised the question as to whether prior studies that included dogs with T-cell lymphoma may underestimate survival times for dogs with B-cell lymphoma treated with single-agent DOX. The objective of this retrospective study was to investigate the outcome of dogs with high-grade B-cell lymphoma treated with DOX and prednisone with or without L-ASP. We hypothesized that these dogs would have an improved overall response rate, progression-free survival (PFS), and overall survival (OS) as compared to previous studies that included dogs with B- and T-cell lymphoma. Identification of factors associated with improved patient outcome was a secondary objective.

## Results

### Patient characteristics

Thirty-three dogs with high-grade B-cell lymphoma treated with at least one dose of DOX between January 2008 and December 2015 were included in the study. Information regarding signalment, weight, stage, and substage is presented in Table 1. Mixed breed dogs (27%) and golden retrievers (12%) were the most common breeds in this study.

**Table 1:** Baseline characteristics of patient population and determination of B-cell immunophenotype (*n* = 33)

Parameter		
Age (years)	Mean ± SD	8.8 ± 2.9
Sex	Male	2 (6%)
	Neutered Male	13 (39%)
	Female	1 (3%)
	Spayed Female	17 (52%)
Weight (kg)	Mean ± SD	27.6 ± 14.2
Breed	Mixed breed	9 (27%)
	Golden retriever	4 (12%)
	German shepherd	2 (6%)
	Scottish terrier	2 (6%)
	Basset Hound	2 (6%)
	Boxer	2 (6%)
	Border collie	2 (6%)
	Other (one each)	10 (31%)
Substage	a	24 (73%)
	b	9 (27%)
Immunophenotype	ICC	21 (64%)
	IHC	5 (15%)
	Flow	7 (21%)

*SD* standard deviation, *ICC* immunocytochemistry, *IHC* immunohistochemistry

Full staging was not performed for all dogs. Thirty-one (94%) dogs had peripheral node involvement, while one dog had thoracic and abdominal lymphadenopathy with no peripheral lymphadenopathy, and one had a colonic mass as the only site of disease. Eleven (33%) dogs were thrombocytopenic and 7 (21%) were anemic prior to first chemotherapy treatment. None of the dogs were hypercalcemic or azotemic. Sixteen (48%) dogs had thoracic radiographs, 15 (45%) dogs had an abdominal ultrasound, and 2 (6%) had a bone marrow aspirate as part of staging. Stage of disease, based on the World Health Organization criteria for canine lymphoma, was not evaluated for association with patient outcome due to the low numbers of dogs with complete staging. Additional sites of disease for the 31 dogs with peripheral node involvement included circulating blasts on initial complete blood cell count (CBC; *n* = 7), pulmonary parenchyma (*n* = 1), and nasal combined with dermal lesions (n = 1). Ten (30%) dogs were substage b and the remaining 23 were considered substage a.

High-grade lymphoma was diagnosed with cytology in 28 dogs (85%) and histopathology in 5 dogs (15%). B-cell phenotype was confirmed using CD79a for immunocytochemistry (ICC) for 21 dogs (64%) and immunohistochemistry (IHC) for 5 dogs (15%) [20]. Seven dogs (21%) had lymph node aspirates submitted for flow cytometric analysis to the Colorado State University Clinical Immunology Laboratory (Fort Collins, CO); B-cell phenotype was determined based on the presence of CD21 ± CD22 on an expanded population of medium to large lymphocytes.

### Treatment

Twenty-one (64%) dogs received L-ASP as induction (400 kg/IU to a maximum of 10,000 units subcutaneously) prior to the first DOX treatment based on clinician preference. The median interval between L-ASP and DOX was 8 days (range, 4–21 days). All dogs received prednisone at least equivalent to 1 mg/kg every 24 h; duration of use and schedule of prednisone tapering was varied.

The median dosage of first doxorubicin was 30 mg/m^2^ (range, 17.5–30 mg/m^2^); 11 dogs weighed < 15 kg and treatment initiated at 1 mg/kg. Seven dogs had DOX therapy initiated at reduced dose, i.e. < 30 mg/m^2^ or < 1 mg/kg for dogs weighing less than 15 kg. The median starting dose for these dogs was 27 mg/m^2^ (range, 25–27 mg/m^2^). The median number of doxorubicin treatments was 5 (range, 1–6 treatments); additional details regarding the number of DOX treatments administered is provided in Table 2. Eighteen (55%) dogs completed the planned treatment protocol, whereas 15 (45%) dogs did not complete their treatment protocol. Of the completed protocol group, 14 dogs completed a 5-treatment protocol, while 4 dogs completed a 6-treatment protocol. For those dogs that did not complete their planned treatment protocol, progressive disease (PD) was determined during the course of treatment in 11 dogs as recorded in their medical record. In the remaining 4 dogs, 3 dogs died either from suspected PD or treatment related toxicities, and for 1 dog the owner elected to discontinue treatment and was lost to follow-up.

**Table 2:** Doxorubicin and prednisone with or without L-asparaginase treatment and patient response

Parameter		
L-asparaginase at induction	Yes	21 (64%)
	No	12 (36%)
Number of doxorubicin treatments	1	7 (21%)
	2	3 (9%)
	3	3 (9%)
	4	1 (3%)
	5	15 (45%)
	6	4 (12%)
Completed protocol	Yes	18 (55%)
	No	15 (45%)
Best response	CR	22 (71%)
	PR	4 (13%)
	SD	2 (6%)
	PD	3 (10%)
	Not evaluated	2

### Outcome

**Fig. 1:**
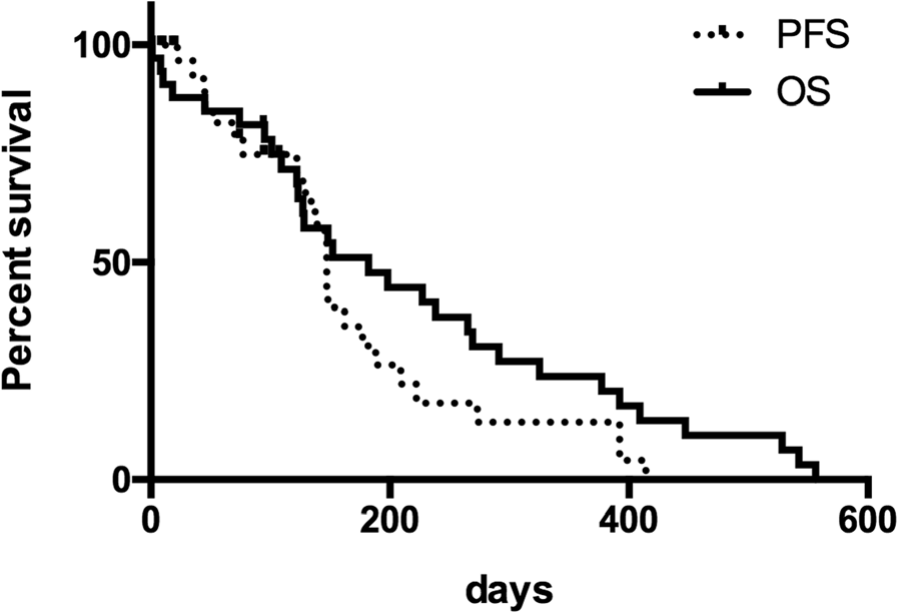
Kaplan-Meier curves of progression free survival (PFS) and overall survival (OS) for 33 dogs treated with doxorubicin and prednisone with or without L-asparaginase for B-cell lymphoma

Thirty-one dogs were able to be assessed for response to treatment. One dog died the day following DOX administration and a second dog died of sepsis secondary to neutropenia 9 days after DOX administration and therefore response could not be assessed but was documented to be in a complete response (CR) at the time of death. The overall response rate (ORR) for these 31 dogs was 84% with 22 (71%) dogs experiencing a CR and 4 (13%) dogs experiencing a partial response (PR) as their best response. There was no difference in response between dogs that received a standard or reduced dose of DOX at initiation (*p* = 1.0). Two dogs had stable disease (SD) and 3 dogs had PD as their best response. The median PFS for all dogs was 147 days (range, 1–414 days; Fig. 1).

The OS for all dogs was 182 days (range, 1–556 days; Fig. 1). Of the 28 dogs able to be offered rescue treatment for progressive disease, 15 dogs received rescue chemotherapy protocols other than prednisone alone. Rescue agents included lomustine (8 dogs), mitoxantrone (6 dogs), COP (3 dogs), vincristine (3 dogs), vincristine/cytarabine/melphalan (3 dogs), dacarbazine (2 dogs), L-ASP (2 dogs), and vincristine/cyclophosphamide/B-cell monoclonal antibody (Aratana Therapuetics, Leawood, KS; 1 dog). Nine dogs continued with prednisone alone at the time of progression. Three dogs were lost to follow-up at a median of 94 days (45–102 days after treatment initiation) and censored at the date of last contact. Two dogs were humanely euthanized due to suspected DOX-induced gastrointestinal toxicity, 4 and 6 days post-infusion. The one-year survival rate for the 30 dogs with known outcomes was 23%, with 23 dogs confirmed dead within a year of treatment initiation. No dogs were alive 2 years after the start of treatment.

Historical pre-treatment prognostic factors such as substage, thrombocytopenia, and body weight were not associated with PFS (Table 3). The only variable associated with improved PFS was protocol completion. The only variables significant for OS were protocol completion and the addition of rescue chemotherapy beyond prednisone alone (Table 3). L-ASP induction was also found not to have affected the PFS (*p* = 0.90), OS (*p* = 0.89), or ORR (*p* = 0.76) between the dogs who received the induction or not. Dogs with substage b lymphoma were not more likely to receive L-ASP than substage a dogs (*p* = 0.26). There was also no difference in PFS or OS for dogs that received 5 versus 6 doses of DOX (*p* = 0.82 and *p* = 0.87, respectively).

**Table 3:** Factors evaluated by univariate analysis for association with progression free survival (PFS) and overall survival (OS)

		n	Median PFS (days)	*p*-value	HR (95% CI)	Median OS (days)	*p*-value	HR (95% CI)
Substage	a	24	147	0.72	1.16 (0.491 to 2.92)	227	0.76	0.89 (0.379 to 2.00)
	b	9	134			148		
L-asparaginase at Induction	No	12	147	0.9	0.95 (0.417 to 2.15)	245	0.89	0.95 (0.458 to 1.96)
	Yes	21	147			152		
Thrombocytopenia	No	22	147	0.18	0.59 (0.210 to 1.30)	198	0.79	0.91 (0.416 to 1.95)
	Yes	11	127			148		
Body weight	< 15 kg	8	147	0.57	0.76 (0.302 to 1.89)	138	0.63	1.219 (0.530 to 2.90)
	> 15 kg	25	147			198		
Anemia	No	7	139	0.55	1.33 (0.540 to 3.35)	152	0.83	0.915 (0.382 to 2.17)
	Yes	26	177			227		
Protocol Completion	No	18	61	< 0.0001	5.66 (17.3 to 324)	101	< 0.0001	4.59 (7.15 to 55.3)
	Yes	15	170			291		
Protocol Duration	5 tx	15	162	0.83	1.12 (0.384 to 3.38)	308	0.87	0.91 (0.289 to 2.83)
	6 tx	4	185			226		
Rescue Chemotherapy	No	13	–	–	–	148	0.013	2.35 (1.36–7.30)
	Yes	15	–			325		

## Discussion

This study sought to determine the response rate, PFS, and OS for dogs with B-cell lymphoma when treated with a DOX and prednisone protocol with or without L-ASP induction; we hypothesized that dogs with B-cell lymphoma would have an improved response rate and outcome as compared to previous studies that included dogs with T-cell forms of lymphoma. Our hypothesis was not supported by our results as this DOX and prednisone protocol, with or without L-ASP induction, resulted in an ORR of 84%, PFS of 147 days, and OS of 182 days. While direct statistical comparison cannot be made, these findings are numerically similar to previously reported DOX protocols when immunophenotype was not assessed (ORR 74–88%; PFS 131–206 days; OS 169.5–295) [15–19]. Our study further supports that a DOX and prednisone protocol continues to result in inferior patient outcome as compared to the PFS and OS previously reported for CHOP (PFS 140–282 days; OS 257–397 days) [1–5]. Completion of protocol was the only variable that was associated with both improved PFS and OS, which is a consequence of improved response to treatment for a subset of patients. No previous historical prognostic factors were found to be associated with outcome, likely due to the evaluation of a more homogenous group of dogs by excluding T-cell immunophenotypes.

It is possible the variations in initially DOX or prednisone dosing could have altered the response rate. We feel this is unlikely, however, as the majority (79%) dogs had treatment initiated at a standard DOX dose of 30 mg/m^2^ (or 1 mg/kg if less than 15 kg) and the median dose was only 10% reduced for those dogs that received a reduced DOX dose at treatment initiation. Additionally, there was no difference in response rates between those two groups. Prednisone is administered orally at home by owners and the tapering schedule was not well documented in most medical records, so the impact of this is challenging to assess. However, the role that prednisone plays in the outcome of dogs with lymphoma when they are treated with a multi-drug chemotherapy protocol without the prednisone has been called into question. There are now two prospective, controlled studies that suggest that prednisone/prednisolone may not affect the response rate, PFS, or OS for dogs with lymphoma [21, 22]. Based on this information, the authors suspect that differences in prednisone dosing and schedule likely have a negligible impact on patient outcome.

An inherent limitation by the retrospective nature of this study is the lack of standardization of response assessment. The Veterinary Cooperative Oncology Group (VCOG) consensus document, describing the evaluation criteria for the treatment response of peripheral nodal lymphoma in dogs is now the standard to measure disease response [23]. Lymph node measurements were not recorded in the medical record for all patients at all visits and therefore response to treatment was based on clinician assessment as recorded in the medical record. It is possible that based on the guidelines, some of the partial responders may have been categorized as having stable disease which could have led to an overestimation of the ORR in this study. However, the VCOG guidelines would not have altered the number of dogs that obtained a CR, as determination of a CR is based on the evaluator’s judgement that lymph nodes have returned to normal size and are non-pathologic [23].

Similarly, toxicity of this protocol was not evaluated in this study due to concern that retrospective assessment of adverse events would be underestimated due to incomplete information in the medical record. However, four dogs were euthanized after their first dose of DOX. One dog with substage b lymphoma died the day following DOX administration, which could have been due to advanced stage of disease or possibly acute tumor lysis syndrome secondary to DOX administration. Two other dogs were euthanized after developing gastrointestinal toxicity 4 and 6 days after DOX treatment. However, both of these dogs were also documented to have progressive disease at this time as well, so it is challenging to elucidate retrospectively how lack of response to treatment may have played a role in the decision to euthanize when gastrointestinal signs secondary to DOX developed. Gastrointestinal toxicity is a common sequala of DOX administration, with up to 2/3 of dogs developing some degree of gastrointestinal toxicosis with < 15% of those considered severe to life-threatening [17, 24, 25]. .A fourth dog was euthanized after becoming septic after DOX-induced toxicity 9 days after treatment. Hematologic toxicities are also commonly reported after DOX administration and while most are mild in nature, they can be severe enough to cause fatal complications as others have reported as well [24, 26].

Higginbotham et al. described a single-agent DOX protocol for the treatment of high-grade B-cell lymphoma, which consisted of an initial induction of 3 treatments administered every 2 weeks with additional DOX treatment at the time of tumor progression [26]. Despite the difference in the timing of DOX administration, our continuous protocol had a comparable median number of treatments to the intermittent treatment protocol (5 treatments vs. 4.5 treatments), as well as similar outcomes (ORR of 78% and median survival time of 169.5 days) [26]. Prospective evaluation of continuous versus intermittent DOX administration could be considered to directly compare the efficacy of these two protocols.

It is possible that the findings in this study were influenced by tumor grade. A distinct biological behavior exists between histologic grades of canine lymphoma, with low-grade lymphoma characterized by slow rate of progression, incomplete responses to DOX-based chemotherapy protocols, and longer survival times [7, 9, 27, 28]. While the distinction of high- and low-grade lymphoma is ideally made based on histopathological evaluation, cytologic assessment of cell size is commonly used in practice to make this determination and has been reported to have a good correlation with histologic samples [29]. Previous studies of single-agent DOX treatment of canine lymphoma have included both high- and low-grade lymphomas when analyzing outcome. Five (12%) dogs treated with single-agent DOX in two previous studies were known to have low-grade lymphoma [15, 19]. The ORR and median survival times of these studies were 76–88% and 237–270 days respectively for all dogs treated. Although including low-grade lymphomas may have negatively impacted the treatment response rate, the median OS outcome was potentially improved. All dogs in the present study had high-grade lymphoma determined by either cytology or histopathology. High-grade B-cell lymphoma, specifically diffuse large B-cell lymphoma, in dogs has an aggressive biologic behavior and when treated with a DOX-based multi-agent chemotherapy protocol as described by Childress et al. has an ORR of 100%, but a relatively short PFI and OS of 252 and 341 days, respectively [10]. The inclusion of dogs with low-grade lymphoma in previous evaluations of single-agent DOX protocols may in part explain why dogs with B-cell phenotype in this study did not have an improved PFS or OS compared to previous studies.

In addition to determining phenotype, flow cytometry can provide cell size and level of antigen expression to further sub-classify lymphomas. A subset of high-grade B-cell lymphomas that have large cells and low MHC class II expression are known to have median survival times similar to high-grade T-cell lymphomas [30]. Only 21% of the dogs in this study were confirmed to have B-cell origin lymphoma via flow cytometry, whereas almost 66% were confirmed by ICC. ICC can be beneficial in conducting direct assessment of cell morphology in combination with its immunoreactivity. However, poor cytologic cellularity or quality can affect ICC interpretation and quantification of antigen expression [31]. Cytologic assessment of cell size is more subjective and MHC class II expression is not routinely assessed with ICC. Therefore, a subset of B-cell lymphomas with poor prognostic factors may have been within this study population, and potentially influencing the PFS and OS negatively. In the future, as additional phenotypic information becomes more readily available to subcategorize high-grade B-cell lymphoma, this could be used to further guide prognosis and treatment recommendations.

Many clinicians routinely initiate treatment for dogs with substage b lymphoma with L-ASP at induction. This practice allows time for owners to contemplate their pets’ diagnosis and to decide on a treatment plan which suits their goals in terms of providing quality of life as well as logistics of finances and time. Dogs receiving L-ASP were included in this study since it has not been found to result in increased remission or survival duration when used at initiation of treatment [5, 32, 33]. Although the use of L-ASP at induction does not influence the outcome of dogs with lymphoma treated with a CHOP protocol, two studies have reported that L-ASP may be useful in the induction phase of treatment and may or may not influence remission and survival times with doxorubicin alone; however, results have been inconsistent [5, 19, 34]. In our study, dogs that did not receive L-ASP had an improved OS when compared to the population receiving L-ASP induction (245 vs. 152 days, respectively); however, this result was not statistically significant. The small sample size of this study precludes robust statistical evaluation of the impact L-ASP has when included at induction with DOX.

The limitations of this study are in part due to its retrospective nature. Inherent with all retrospective studies, some dogs were lost to follow-up and censored from OS analysis (*n* = 3), and complete staging was not performed in most dogs. Prednisone was administered in addition to the DOX and could have led to an over estimation of response rate. Many protocols incorporate prednisone in the induction period to improve patients’ quality of life, and in this study the remission duration and survival exceeded the known outcomes associated with prednisone alone [9]. Inconsistent rescue therapy and choice of euthanasia have an overall impact on OS, especially if time and financial considerations are important factors. Lastly, the small sample size could have impeded relevant statistical analysis.

## Conclusions

A DOX and prednisone protocol, with or without L-ASP induction, for the treatment of canine high-grade B-cell lymphoma has similar response rates, PFS, and OS to previously published protocols that did not assess for immunophenotype. While less efficacious than multi-agent protocols for lymphoma, this protocol may be suitable for owners unable to pursue a more time or financially intensive protocol and who seek therapeutic benefit greater than prednisone alone.

## Methods

### Study population

Dogs diagnosed with high-grade B-cell lymphoma and treated with at least one dose of DOX were retrospectively identified through the University of California, Davis Veterinary Medical Teaching Hospital, Oregon State University Lois Bates Acheson Veterinary Teaching Hospital, and Arizona Veterinary Oncology medical record databases from January 2008 to December 2015. Inclusion criteria comprised a diagnosis of high-grade lymphoma with measurable disease present at treatment initiation, confirmed B-cell phenotype, and intent to treat with DOX chemotherapy with no prior cytotoxic chemotherapy.

Signalment, body weight, age at diagnosis, clinical signs at presentation, and pretreatment diagnostics, such as CBC, serum chemistry panel, urinalysis, thoracic radiographs, abdominal imaging, and bone marrow evaluation, were abstracted from the medical record for each dog. Lymph node cytology, histopathology, and/or reports were reviewed to confirm reporting of large cell morphology and to establish the method used for immunophenotype determination.

### Treatment

Dogs that had previously been treated with chemotherapy were excluded; however, oral prednisone treatment of ≤14 days and L-ASP induction ≤21 days prior to DOX administration were acceptable. Each dog received at least one dose of DOX administered intravenously (30 mg/m^2^ for dogs > 15 kg or 1 mg/kg for dogs ≤15 kg). Subsequent DOX treatments were administered every 3 weeks for five to six total treatments unless progressive disease occurred prior to completion of the prescribed protocol.

Information obtained regarding the treatment protocol included use of L-ASP induction, number of treatments, and whether the DOX protocol was completed. Rescue protocols were recorded in cases where relapse occurred, and the owners elected additional therapy. Follow-up information was obtained from medical records and by phone calls to the referring veterinary hospitals. Factors evaluated for association with PFS and OS included substage, body weight, anemia, thrombocytopenia, hypercalcemia and/or azotemia at the time of diagnosis, number of total treatments (5 versus 6), protocol completion, and use of L-ASP at treatment induction. Patient outcome criteria included maximal response to therapy, PFS, and OS. A CR was considered as resolution of all clinically detectable disease. A designation of PR, SD, or PD was determined based on clinician assessment in the medical record as lymph node measurements were not recorded in all cases and standard VCOG criteria for nodal lymphoma could not be retrospectively applied to all cases. Responses and stable disease needed to be maintained for a minimum of 21 days.

### Statistical analyses

Continuous data were tested for normality using the D’Agostino-Pearson test and reported using mean or median and range; categorical data were reported as frequencies and percentages. ORR were defined as the number of dogs experiencing partial or complete remission divided by the total number of dogs treated. Chi-square analysis was used to evaluate association between use of L-ASP at induction and tumor response (CR, PR or SD/PD). Fisher’s exact test was used to evaluate association between substage and use of L-ASP at induction as well as association between starting DOX dosage (standard versus reduced) and response (CR/PR versus SD/PD). PFS was defined as the time of first treatment with either L-ASP or DOX until progression of disease. OS was defined as the time from first treatment with either L-ASP or DOX and death from any cause. PFS and OS analyses were performed using the Kaplan-Meier product limit method. Dogs were censored at the date of last contact if they were still alive at the time of analysis or last follow-up. A *p*-value of ≤0.05 was considered statistically significant. A commercially available software program was used to perform statistical analyses (Prism v 6.0b, GraphPad Software, La Jolla, CA).

## Abbreviations

CBC: Complete blood count
CHOP: Cyclophosphamide, doxorubicin, vincristine, prednisone
CR: Complete response
DOX: Doxorubicin
ICC: Immunocytochemistry
IHC: Immunohistochemistry
L-ASP: L-asparaginase
ORR: Overall response rate
OS: Overall survival
PD: Progressive disease
PFS: Progression free survival
PR: Partial response
SD: Stable disease
VCOG: Veterinary Cooperative Oncology Group
